# Liver Restores Immune Homeostasis after Local Inflammation despite the Presence of Autoreactive T Cells

**DOI:** 10.1371/journal.pone.0048192

**Published:** 2012-10-24

**Authors:** Kathie Béland, Pascal Lapierre, Idriss Djilali-Saiah, Fernando Alvarez

**Affiliations:** 1 Division of Gastroenterology, Hepatology and Nutrition, Centre Hospitalier Universitaire Sainte-Justine, Montréal, Quebec, Canada; 2 Microbiology and Immunology department, University of Montreal, Montréal, Quebec, Canada; 3 Department of Pediatrics, University of Montreal, Montréal, Quebec, Canada; Albert Einstein Institute for Research and Education, Brazil

## Abstract

The liver must keep equilibrium between immune tolerance and immunity in order to protect itself from pathogens while maintaining tolerance to food antigens. An imbalance between these two states could result in an inflammatory liver disease. The aims of this study were to identify factors responsible for a break of tolerance and characterize the subsequent restoration of liver immune homeostasis. A pro-inflammatory environment was created in the liver by the co-administration of TLR ligands CpG and Poly(I:C) in presence or absence of activated liver-specific autoreactive CD8^+^ T cells. Regardless of autoreactive CD8^+^ T cells, mice injected with CpG and Poly(I:C) showed elevated serum ALT levels and a transient liver inflammation. Both CpG/Poly(I:C) and autoreactive CD8^+^T cells induced expression of TLR9 and INF-γ by the liver, and an up-regulation of homing and adhesion molecules CXCL9, CXCL10, CXCL16, ICAM-1 and VCAM-1. Transferred CFSE-labeled autoreactive CD8^+^ T cells, in presence of TLR3 and 9 ligands, were recruited by the liver and spleen and proliferated. This population then contracted by apoptosis through intrinsic and extrinsic pathways. Up-regulation of FasL and PD-L1 in the liver was observed. *In conclusion,* TLR-mediated activation of the innate immune system results in a pro-inflammatory environment that promotes the recruitment of lymphocytes resulting in bystander hepatitis. Despite this pro-inflammatory environment, the presence of autoreactive CD8^+^ T cells is not sufficient to sustain an autoimmune response against the liver and immune homeostasis is rapidly restored through the apoptosis of T cells.

## Introduction

The liver anatomical position between the splanchnic venous system and the systemic circulation exposes the organ to food, flora and self-antigens to which immune reactivity must be avoided. However, the liver must also protect itself against potentially harmful pathogens and allow the development of effective immune responses. This delicate balance between tolerance and immunity is crucial to the integrity of the liver. A disturbed immune homeostasis could lead to autoimmune liver diseases such as autoimmune hepatitis (AIH) or primary biliary cirrhosis or the opposite, chronic viral infections such as hepatitis B and C virus infections.

It is currently hypothesized that a viral infection could contribute to the initiation of a pathological autoimmune reaction in predisposed individuals with circulating liver-specific autoreactive lymphocytes [1]. It has been postulated that at least two independent steps are needed to induce an autoimmune reaction against the liver [2]. First, the presence of functional activated autoreactive T cells. Second, a favorable local microenvironment induced either by liver-tropic pathogens or Toll-like receptors (TLR) stimulation through microorganism constituents. Recently, control of the immuno- privileged status of the liver was attributed to TLR3 stimulation [3]. Liver damage following transfer of autoreactive T cell was observed only in presence of a concomitant virus infection or stimulation of the innate immune system by Poly(I:C) and CpG [3].

**Figure 1:**
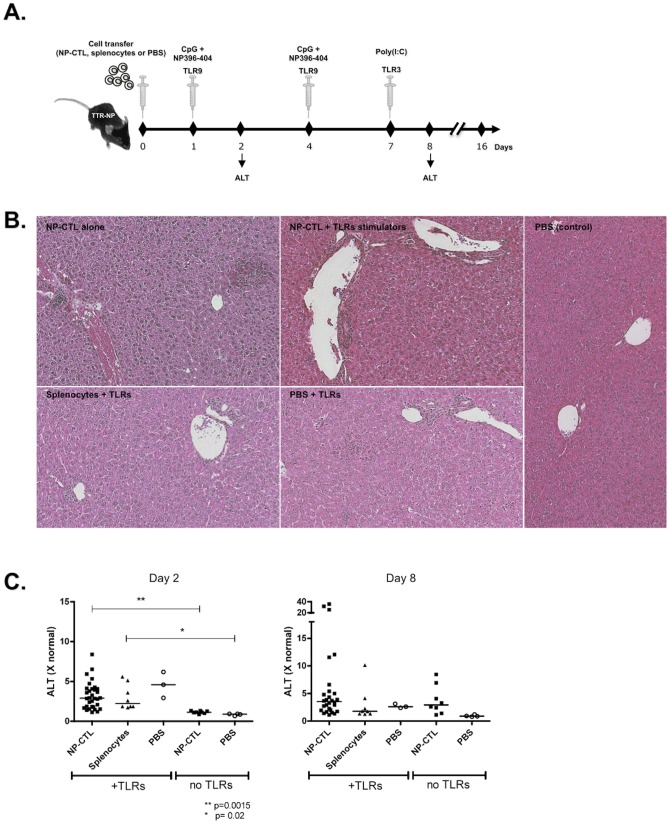
Liver Inflammation Caused by TLR9 and TLR3 Stimulation after Autoreactive CD8^+^ T cells Transfer Contributes to Liver Injury. **A**) Protocol for NP396–404 specific CD8^+^T (NP-CTL) cells transfer and TLRs stimulation. **B**) Representative liver histology under various conditions (day 8). **C**) Serum ALT levels were elevated on day 2 and 8 when NP-CTL were transferred along with Poly(I:C) and CpG. Syngenic splenocytes transfer along with TLR3 and 9 stimulation or TLRs stimulation alone resulted in similar elevation of serum ALT. Horizontal lines represent median values, TLRs: TLR3/9 stimulation.

TLR3 stimulation by Poly(I:C) is known to induce liver damage, promoting inflammation of the liver through an increased expression of Vascular cell adhesion molecule-1 (VCAM-1) [4]. Stimulation of TLR3 has also been linked to autoimmune liver disease as repeated injection of Poly(I:C) can mimic a disease similar to primary biliary cirrhosis [5]. However, these effects are dependant on the sustained administration of Poly(I:C). Similarly, TLR9 stimulation by CpG has also been linked to a break of liver tolerance [6], [7]. In a model of heart and liver transplant, CpG injections precluded spontaneous hepatic tolerance [6]. Moreover, repeated CpG injections can cause a transient disease similar to autoimmune hepatitis, resolving with the end of CpG administration [7]. Therefore, TLR3 and TLR9 stimulation can result in a pro-inflammatory environment in the liver, detrimental to the maintenance of tolerance. However, it is not known if a TLR3 and 9-induced pro-inflammatory environment in presence of circulating autoreactive T cells (similar to a viral bystander hepatitis in a predisposed individual) is sufficient to break tolerance and lead to a chronic autoimmune liver disease. The main aim of this study was to identify factors responsible for liver immune homeostasis. To achieve this, a transgenic mouse known to be permissive for the development of a chronic autoimmune liver disease [8] was used to ensure that no other limiting factors for the development of a persistent autoimmune response, central tolerance for example, were present.

**Figure 2:**
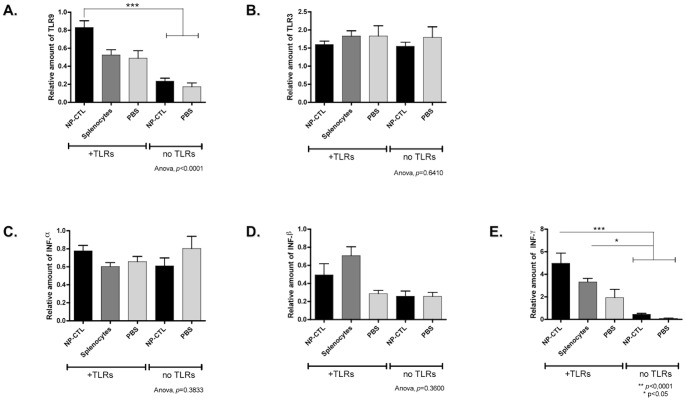
Effect of CpG and Poly(I:C) on the Expression of TLRs and Interferon in the Liver. TLR9 (**A**), but not TLR3 (**B**) (*p* = 0.6410), was over-expressed when NP-CTL were transferred along with CpG and Poly(I:C) (ANOVA Tukey Post-hoc test, *p*<0.0001 compared to no TLR stimulation). **C**) Interferon α, (**D**) β and (**E**) γ expression were quantified. Only INF-γ was over-expressed when NP-CTLs were transferred along with TLR stimulation or, to a lesser extent, when syngenic splenocytes were transferred and TLRs were stimulated (ANOVA Tukey Post-hoc test, INF-α *p* = 0.3833, INF-β *p* = 0.3600, INF-γ *p*<0.0001 and *p*<0.05 compared to TLR stimulation). Means ^+^/− SEM are depicted.

Herein, we show that while a pro-inflammatory environment and liver-specific autoreactive T cells can induce a transient break of tolerance, they were not able to induce persistent liver damage. TLR3 stimulation was not sufficient to sustain a break of tolerance against a liver autoantigen, as immune homeostasis in this organ was rapidly restored through the induction of T cell apoptosis.

**Figure 3:**
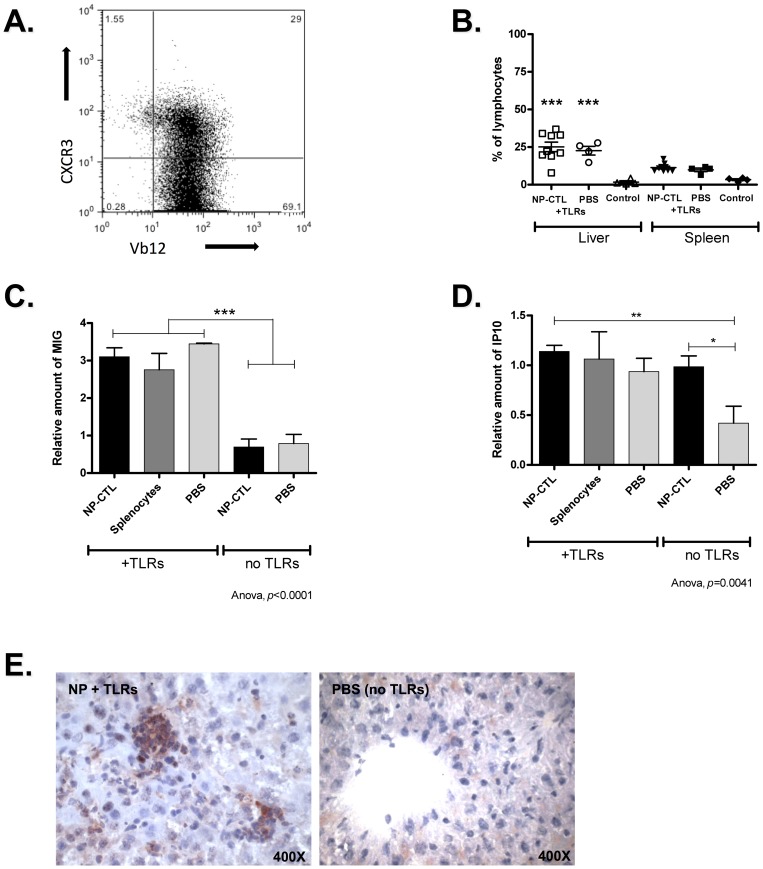
CXCR3 is involved in the Homing of Lymphocytes to the Liver. **A**) 29% of NP-CTL cells were CXCR3^+^ before transfer (gated on CD3^+^ cells). **B**) There were significantly more CXCR3^+^ cells among CD3^+^ T cells in liver-infiltrating lymphocytes (LIL) of mice transferred with NP-CTL and TLR3 and 9 stimulation or with TLRs stimulation alone compared to untreated mice (ANOVA Tukey post-hoc test, *p*<0.0001). This enrichment of CXCR3^+^ T cells was not observed in the spleen. Control mice were TTR-NP mice that did not received any treatment. **C**) MIG (CXCL9) was over-expressed in the liver in response to TLR3 and TLR9 stimulation (ANOVA Tukey post-hoc test, *p*<0.0001). **D**) IP-10 (CXCL10) was over-expressed in the liver in response to TLR3 and 9 stimulation (ANOVA Tukey post-hoc test, *p* = 0.0029). **E**) Immunohistochemistry showing MIG liver expression in a mouse that received NP-CTL, CpG and Poly(I:C) (400x). Means ^+^/− SEM are depicted.

## Experimental Procedures

### NP-specific CTL clones and TLR stimulation

TTR-NP mice (C57BL/6 background) [8], expressing the nucleoprotein (NP) from the lymphocytic choriomeningitis virus (LCMV) exclusively in hepatocytes under control of the transthyretin (TTR) liver-specific promoter region, received NP-specific, H-2D^b^-restricted, CD8^+^ CTL clones (NP-CTL) recognizing the immunodominant epitope NP396–404 (FQPQNGQFI) (NP18 clone; kindly provided by M.B.Oldstone) [9]. The NP18 clone [9] (NP-specific T cells) bears a TCR Vβ12 chain (Djilali-Saiah I., manuscript submitted). NP-CTLs, either NP18 T cell clone or cells harvested from the spleen and lymph nodes of TNP4 mice (NP18-TCR-tg mice on C57BL/6 background) (Djilali-Saiah I., manuscript submitted), were propagated in culture using feeders cells coated with the NP396-404 peptide and IL-2 (40U/mL, Invitrogen, CA). Transfer of NP-CTL from TNP4 mice or the NP18 clone resulted in similar results in all experiments. Cells (10×10^6^) were injected intravenously in the tail vein of 10–12 weeks-old TTR-NP female mice on day 0 (Figure 1 a). When mentioned, transferred cells were labeled with 5 μM CFSE (Molecular Probes, CA) to trace cells *in vivo*. In order to replicate a bystander hepatitis (liver pro-inflammatory environment) in an individual with circulating autoreactive T cells, CpG (TLR9 stimulation) and Poly(I:C) (TLR3 stimulation) were administered to NP-CTLs-transferred mice. CpG1826 (20 μg,GACGTT) (Hycult biotechnology b.v., Netherlands) and NP396–404 peptide (10 μg) in saline buffer were injected IP on day 1 and 3 (Figure 1 a). Poly(I:C) (500 μg, Sigma-Aldrich, MO) was then IP injected on day 7. Unless otherwise mentioned, mice were sacrificed on day 8 (Figure 1 a). Control TTR-NP mice received unstimulated syngeneic splenocytes and/or buffer vehicle instead of TLR stimulators. Animals were kept in a pathogen-free facility and were treated according to the Canadian Council on Animal Care (CCAC) guidelines. The protocol was approved by the *Comité Institutionnel de Bonnes Pratiques Animales en Recherche* of CHU Sainte-Justine.

**Figure 4:**
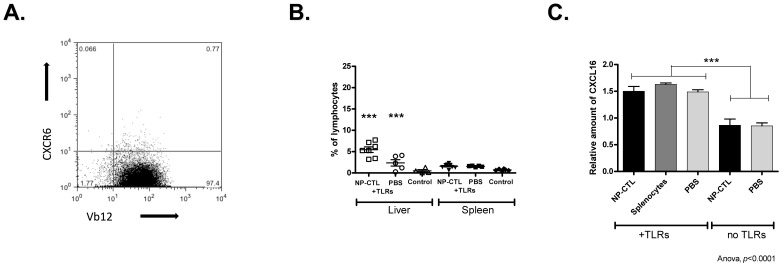
CXCR6 Involvement in the Homing of Lymphocytes in the Liver. **A**) NP-CTLs were mainly CXCR6^-^ before transfer. **B**) A significant increase of CXCR6^+^ CD3^+^ T cells was observed in the liver when NP-CTL cell were transferred along with TLRs stimulation (6.8%±4.1, *p*<0.0001) or, to a lesser extent, with TLRs stimulation alone (2,3%±1,6, *p*<0.0001) compared to controls (no treatment). The number of CXCR6^+^ CD3^+^ T cells in the spleen was not affected by either treatment. Control mice were TTR-NP mice that did not received any treatment. **C**) CXCL16 was over-expressed in the liver in response to TLR3 and TLR9 stimulation (*p*<0.001) as measured by RT-PCR. Means ^+^/− SEM are depicted.

### Serum ALT Activity

Serum alanine aminotransferase (ALT) levels were measured in a Beckman-Synchron CX9 apparatus, from blood taken on day 2, 8 and 16 post-transfer.

**Figure 5:**
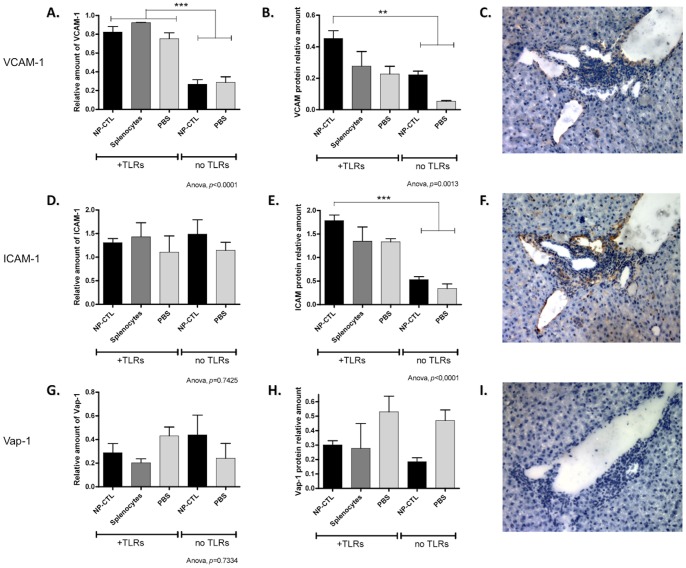
Adhesion Molecules VCAM-1 and ICAM-1, but not Vap-1, are Up-regulated in Response to Autoreactive Cells and TLRs Stimulation. Levels of specific mRNAs (**A, D, G**) and proteins expression (**B, E, H**) in the liver were measured for VCAM-1 (**A–C**), ICAM-1 (**D–F**) and Vap-1 (**G–I**). Only VCAM-1 mRNA was up-regulated in response to TLRs stimulation (ANOVA, *p*<0.0001). At the protein level, VCAM-1 (*p = *0.0013) and ICAM-1 (*p*<0.0001) were over-expressed when NP-CTL were transferred along with TLRs stimulation. Protein expression was confirmed by immunohistochemistry (**C, F, I**). Means ^+^/− SEM are depicted.

### Histopathology and TdT-mediated dUTP-biotin Nick-End Labeling (TUNEL)

Mice were sacrificed on day 1, 3 and 8 post-transfer; liver sections were dehydrated, embedded in paraffin, sectioned, and stained with Hematoxylin-Phloxin-Safran. To perform TUNEL assay, 7 μm cryopreserved liver section were fixed in 1% paraformaldehyde. TUNEL assay was performed using the ApopTag Peroxidase Kit (Chemicon International, CA) following manufacturer's instructions. Data are expressed as the mean number of TUNEL-positive cells in five different 400X-magnified field ^+^/− SEM.

**Figure 6:**
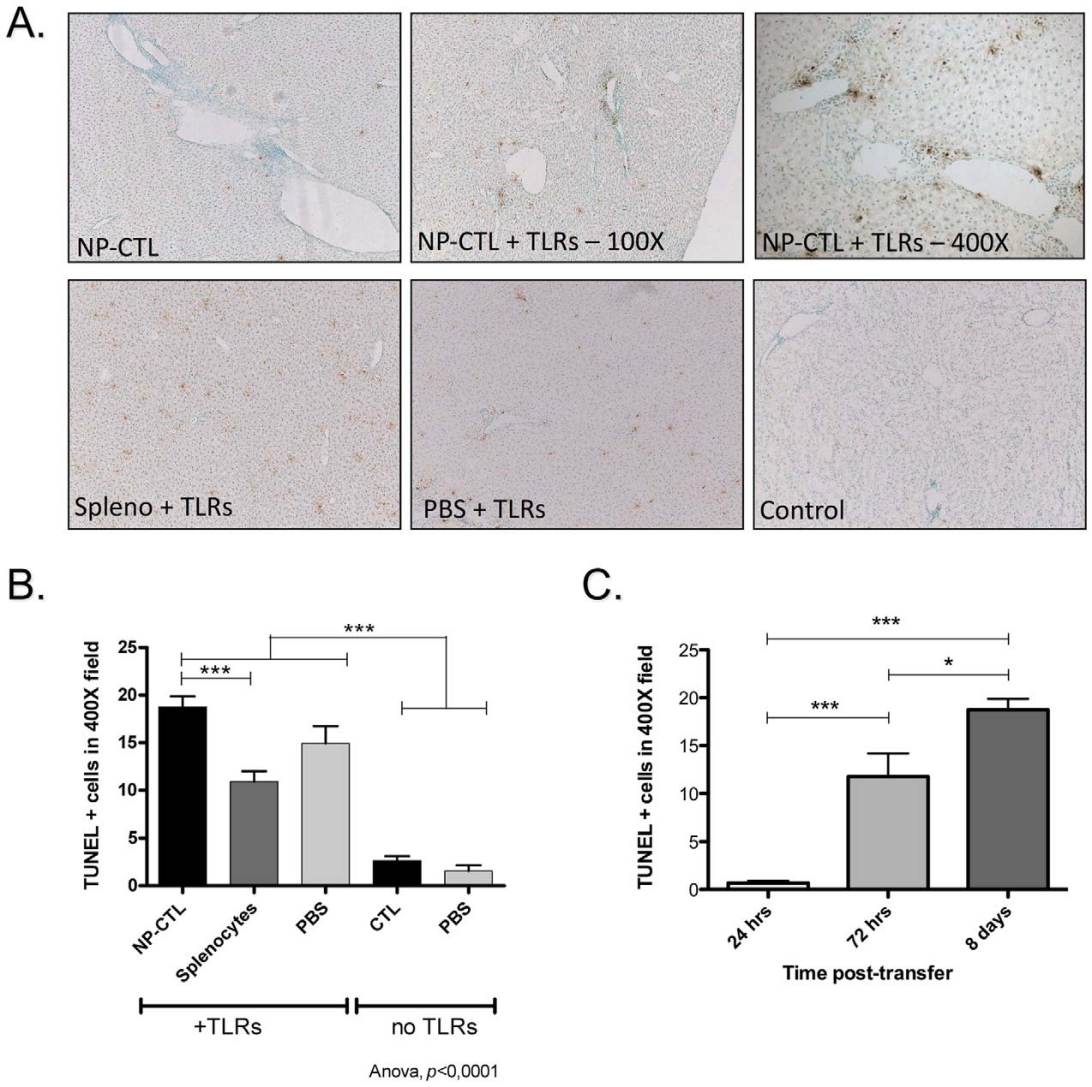
Apoptosis in the Liver. **A**) TUNEL staining revealed apoptotic cells among hepatocytes and lymphocytes in liver from mice that received CpG and Poly(I:C), with or without autoreactive CTLs. **B**) TUNEL positive cells were more frequent in liver from mice that received NP-CTLs with TLR3 and 9 ligands or TLR ligands alone (ANOVA, Tukey post-hoc test, *p<*0.0001). **C**) Kinetic of apoptosis in the liver. A significant increase in the number of TUNEL^+^ cells in the liver was observed 72 hrs post-transfer (compared to 24 hrs), 2 days after TLR9 first stimulation. A significant increase was also observed 8 days after transfer (*p<0.05, ***p<0.001, ANOVA with Tukey post-test). Data are expressed as means of TUNEL-positive cells in a 400 high power field ^+^/− SEM.

### Lymphocytes Isolation

Liver-infiltrating lymphocytes (LIL) were isolated from perfused liver as previously described [10]. Lymphocytes from blood and spleen, lymph nodes (caudal, lumbar, inguinal, axillaries and brachial) and lungs were isolated by finely mincing the tissues in RPMI1640, which were then passed through a 100-gauge mesh and centrifuged at 1,500 g for 5 min.

**Figure 7:**
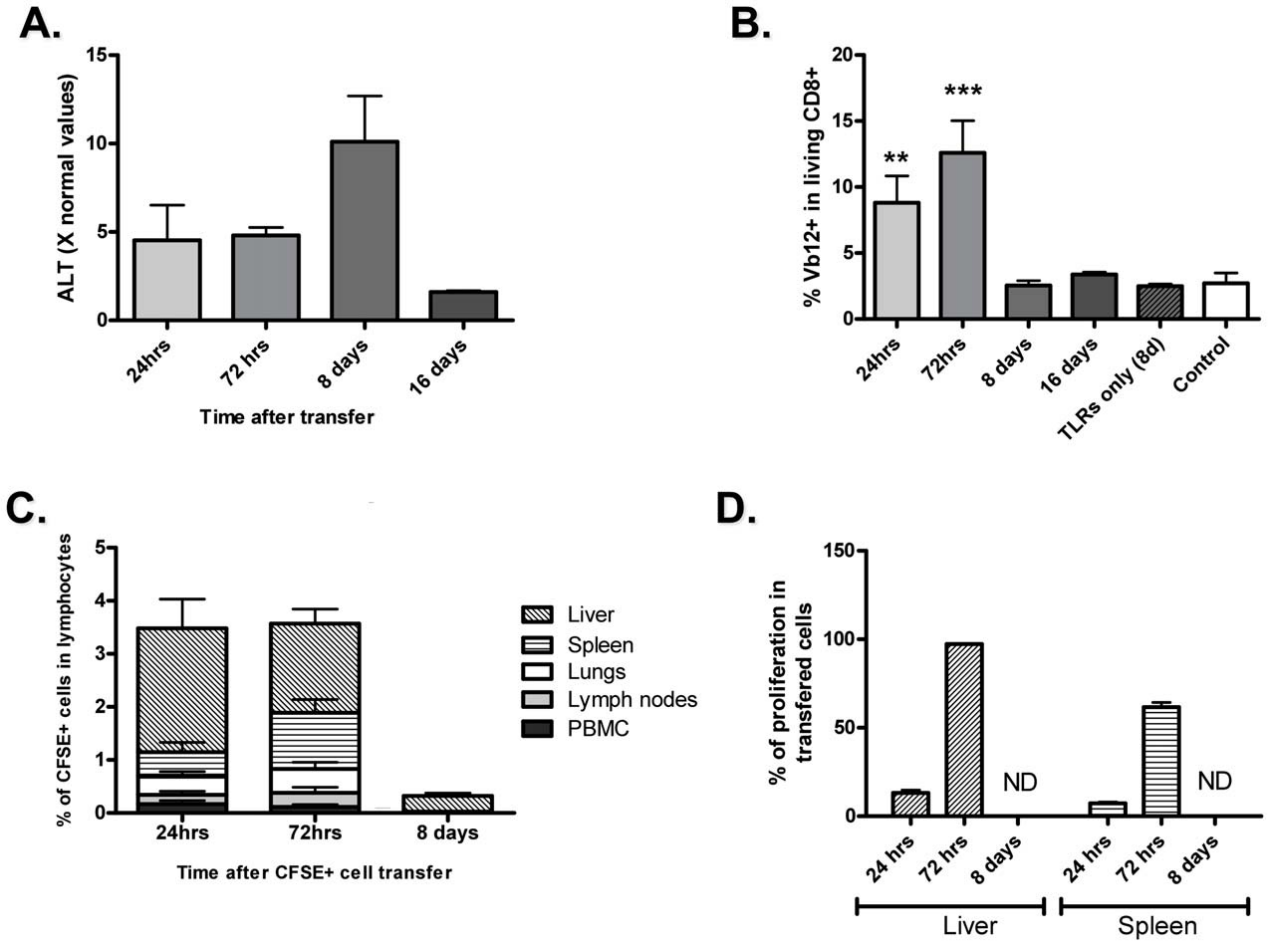
Restoration of Tolerance. **A**) ALT levels returned to normal 16 days after adoptive transfer of NP-CTL and TLR3 and 9 stimulation. **B**) The population of autoreactive T cells directed against NP (CD8^+^Vb12^+^ cells), among liver-infiltrating lymphocytes, increased 24 hrs post-transfer (*p<*0.01) and continued to increase until 72 hrs, when TLR stimulators were administered (*p<*0.0001), but this population declined afterwards and returned to levels found in mouse that did not received autoreactive CTLs (controls). **C**) CFSE^+^ transferred cells were found mainly in the liver, but also in the spleen, on day 1 and 3 after adoptive transfer. However, CFSE-labeled cells were found in very low proportion at 8 days after transfer. **D**) *In vivo* proliferation of transferred lymphocytes was observed at 24 hrs and 72 hrs post-injection but was not detectable at 8 days. Percentage of cells that underwent at least one division among the retrieved CFSE-labeled transferred cells is represented. Means ^+^/− SEM are depicted.

### Flow Cytometry Analysis

The following antibodies/reagent were used for flow cytometry analyses: NP396–404-specific tetramers (Tet-NP396 PE, kindly provided by A. Lamarre, INRS-Institut Armand-Frappier), anti-mouse CD3 FITC, anti-mouse CD8 APC, anti-mouse CD69 FITC, anti-mouse CXCR3 PerCP, anti-mouse PD-1 FITC (eBiosciences, CA), anti-mouse CXCR6 APC (R&D Systems, MN), anti-mouse Vb12 PE (Biolegends, CA) and 7-AAD for viability staining (BD Biosciences, Canada). Apoptosis was assessed using FAM-FLICA *in vitro* kit for Caspase 8 and 9 (ImmunoChemistry Technologies, MN), Annexin V PE labeling (Bender MedSystems, CA) and 7-AAD staining (BD Biosciences, Canada).

**Figure 8:**
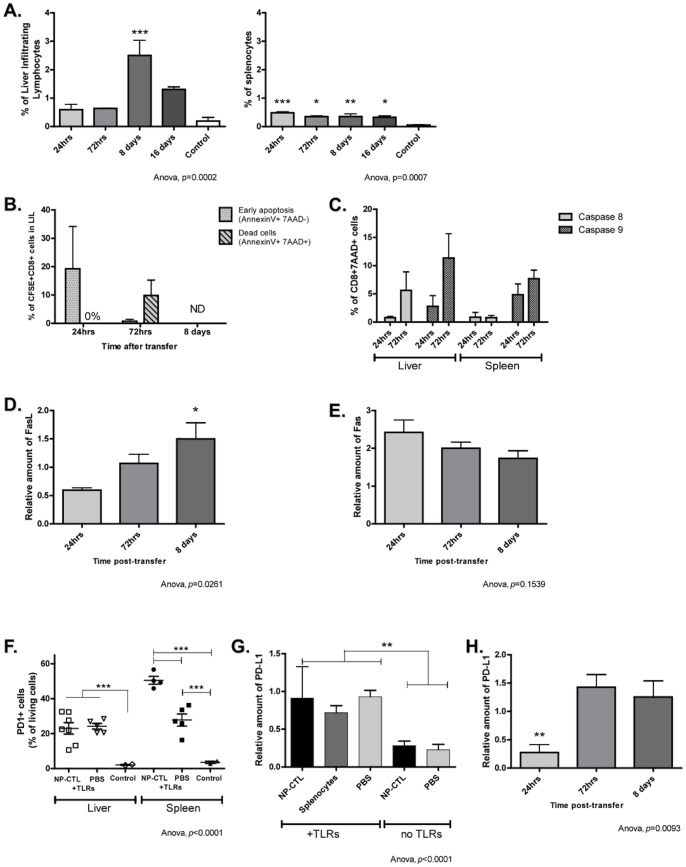
Liver Protection Against Inflammation Through the Contraction of Autoreactive CD8^+^ T Cells. **A**) A contraction in the number of CD8^+^ T cells occurred (7-AAD^+^CD8^+^cells) in both the liver (*p* = 0.0002) and spleen (*p* = 0.007) of NP-CTL transferred mice injected with TLR3 and 9 ligands. 7-AAD^+^ CD8^+^ T cells were mainly found in the liver and were in higher proportion on day 8 (*p* = 0.0002). Data are expressed as means of % of 7-AAD^+^ CD8^+^ T cells among lymphocytes ^+^/− SEM, * in reference to TTR-NP control mice (no treatment). **B**) CFSE^+^ cells found in the liver of NP-CTL transferred mice with TLR3 and 9 stimulation at 24 hrs were not dead (Annexin V^+^ 7-AAD^+^) but 19,3% were in a state of early apoptosis (Annexin V ^+^). At 72 hrs, 9,9% were dead (Annexin V^+^ 7-AAD^+^). No CFSE^+^ cells were found at 8 days (one day after Poly(I:C) administration). **C**) Harvested 7-AAD^+^ CD8^+^ T cells from spleen and liver had activated caspases 8 and 9. More caspase 9^+^ cells than caspase 8^+^ cells were retrieved in the spleen while both caspases were activated in the liver at 72 hrs. **D**) FasL was up-regulated in the liver of mice transferred with NP-CTLs after TLR3 and 9 stimulation and this up-regulation was increased as tolerance was restored (24 hrs<72 hrs<day 8); while (**E**) Fas receptor expression remained unchanged in the liver during this period (*p* = 0.1539). **F**) Significantly more cells from the liver and spleen of NP-CTL transferred mice injected with TLR3 and 9 ligands expressed PD-1 on day 8 after transfer. This up-regulation was induced by the administration of CpG and Poly(I:C) and was more pronounced when NP-CTLs were transferred. Control mice were TTR-NP mice that did not received any treatment. **G**) PD-L1 was over-expressed in the liver in response to TLR3 and TLR9 stimulation (ANOVA, *p<*0.0001) and (**H**) its expression peaked 72 hrs after transfer and remained stable until day 8. Means ^+^/− SEM are depicted.

### Immunohistochemistry

7μm cryopreserved liver section were fixed in methanol. Slides were quenched in 0.2% H_2_O_2_ and incubated with anti-mouse Inter-Cellular Adhesion Molecule 1 (ICAM-1) (1 ∶20), anti-mouse VCAM-1 (1∶20), anti-mouse MIG (monokine induced by INF-γ, CXCL9)(1∶50) (R&D Systems, MN) or anti-mouse Vascular adhesion protein 1 (Vap-1) (1∶50, SantaCruz, CA). Peroxidase-conjugated secondary antibodies (Biosource International, CA) were used at 1∶400 and detected with AEC^+^ High sensitivity chromogen substrate (DakoCytomation, Denmark).

### RNA Isolation and RT-PCR

Total RNA from excised liver was prepared using the Qiagen RNeasy kit (Qiagen, CA). A DNA digestion was performed on each sample with RNAse-Free DNAse (Qiagen, CA). Expression levels were measured using specific primers for: *1*) *TLRs*: TLR3 [11], TLR9 [12]; *2) Cytokines*: INF-α [11], INF-β, INF-γ [13]; *3) Adhesion molecules*: ICAM-1 [14], VCAM-1 [15], Vap-1; *4) Chemokines binding CXCR3*: MIG (CXCL9) [16], IP-10 (INF-γ inducible protein, CXCL10); *5) Chemokine binding CXCR6:* CXCL16 [17]; *6) Immunomodulators:* FasL [18], Fas and Programmed Death ligand 1 (PD-L1) [19]. PCRs were performed using the One-Step RT-PCR kit (Qiagen, CA) and murine β-actin was used as internal reference. Specific primers are listed in Table S1.

### Mouse Liver Homogenate and Western Blot Analysis

Mice livers were homogenized as described [10]. Protein expression levels of ICAM-1 (1∶3,000), VCAM-1 (1∶3,000) (R&D Systems, MN) and Vap-1 (1∶200, SantaCruz, CA) were characterized by western blot using NP as internal control (rabbit anti-NP serum 1∶2,000). Peroxidase-conjugated secondary antibodies were used at 1∶20,000 (Biosource International, CA) and detected with chemiluminescence blotting substrate (Roche Applied Science, IN). Quantification was performed with Gel-Pro Analyser 3.1 software.

### Statistical Analysis

All statistical analyses were performed using GraphPad Prism 4. Differences were considered significant at *p*<0.05. Only two-tailed analyses were performed. When performing One-way ANOVAs, a Tukey's post-hoc test was performed, and for Two-way ANOVAs, a Benferroni post-hoc test was used.

## Results

### TLR3 and 9 Stimulation Induces Bystander Hepatitis and Promotes Liver Injury

To avoid non-optimal activation of naive T cell by the liver [20], NP-specific T cells were activated and propagated *in vitro* with IL-2 and NP396–404 peptide. Autoantigen specificity was assessed by flow cytometry; 92,9%±4,8 of viable injected CD8^+^ T cells were specific for the NP.

Mice that received autoreactive NP-CTLs cells alone or in combination with TLR3 and 9 stimulation, or TLRs stimulation alone, presented similar grades of liver inflammation on day 8 (Figure 1 b). Injection of CpG to TTR-NP mice with or without previous NP-CTL transfer resulted in an early (day 2) significant increase in serum ALT level (Figure 1 c) (*p* = 0,0015 compared to NP-CTL transfer alone, and *p* = 0.02 between CpG and PBS injections in absence of NP-CTL transfer). This increase in serum ALT level was still observed 8 days after transfer (Figure 1 c). CpG, NP396–404 peptide and Poly(I:C) injections alone or after syngeneic cells transfer resulted in a similar liver inflammation and injury (Figure 1 c). Therefore, CpG and Poly(I:C) injections were sufficient to induce a bystander hepatitis.

### CpG and Poly(I:C) Administration and Liver Inflammatory Environment

TLR9 expression was increased in the liver in response to autoreactive NP-CTL transfer and TLRs stimulation (*p*<0.0001, Figure 2 a). The expression of TLR3 remained the same when autoreactive CTLs were transferred with or without TLR3 and 9 stimulation (*p* = 0.6410, Figure 2 b).

The expressions of interferon-α, β and γ (INF) in the liver were studied in response to TLRs ligands (Figure 2 c-e). Among them, only the expression of interferon-γ was significantly up-regulated (*p*<0.0001). Autoreactive NP-CTLs did not influence interferon-γ production on day 8 post-transfer. TLRs stimulation was responsible for the observed INF-γ up-regulation since the transfer of control cells with TLR3 and 9 stimulation also increased its expression (*p*<0.05).

### TLR 3 and 9 Stimulation Promotes Homing of Lymphocytes to the Liver

Lymphocyte homing to the liver is modulated by chemokines such as CXCR3 and CXCR6 ligands [21], [22]. Before transfer, NP-CTLs were mostly CXCR3^-^ and CXCR6^-^ (Figure 3 a and 4 a). Eight days post-transfer, a significantly higher proportion of CXCR3^+^ (*p*<0.0001) and CXCR6^+^ (*p*<0.0001) T cells were found among liver infiltrating lymphocytes (Figure 3 b and 4 b). This enrichment was caused by TLR3 and 9 stimulation since it was also observed in mice that did not received autoreactive T cells. It was observed in the liver but not in the spleen (Figure 3 b and 4 b). This enrichment was not the direct consequence of adoptive cell transfer since the majority of transferred NP-CTL were CXCR3^−^ and virtually all were CXCR6^-^ before transfer.

Expressions of CXCR3 ligands, MIG (CXCL9) and IP-10 (CXCL10), in the liver were evaluated 8 days after NP-CTL transfer and/or TLR3 and 9 stimulation (Figure 3 c–d). MIG was up regulated in response to TLR stimulation only (*p*<0.0001). Interestingly, IP-10 was over-expressed in response to NP-CTL transfer or TLRs stimulation (*p* = 0.0041, Figure 3 d). The simultaneous presence of both stimuli did not have a synergistic effect (Figure 3 d). MIG over-expression was confirmed by immunohistochemistry (Figure 3 e). These results are similar to the increased IP-10 and MIG expression observed in mice after Poly(I:C) injection alone [3]. The expression of CXCL16, the CXCR6 ligand, was up-regulated in response to CpG and Poly(I:C) administration (*p<*0.0001, Figure 4 c) but was not influenced by the presence of autoreactive T cells. Therefore, stimulation of TLR3 and 9 promotes homing of CXCR3^+^ and CXCR6^+^ T cells, including autoreactive T cells, resulting in bystander hepatitis.

Chemokines and INF-γ mRNA levels in the liver were measured at 3 time-points after cell transfer: 24 hrs, 72 hrs and 8 days (Figure S1). Interferon-γ expression was up regulated on day 3 post-transfer. Expression levels of IP-10 and MIG increased rapidly on day 3 and slowly decreased afterwards (IP-10, *p*<0.0001 24 hrs vs 72 hrs and 72 hrs vs 8 days; MIG, *p*<0.0001 for 24 hrs vs 72 hrs or 8 days and *p*<0.001 for 72 hrs vs 8 days). CXCL16 expression slowly increased over time and was significantly higher on day 8 (*p*<0.0001).

### Recruitment of Lymphocytes to the Liver

To study the recruitment of lymphocytes to the liver, VCAM-1, ICAM-1 and Vap-1 expression levels were quantified. VCAM-1 mRNA levels were up-regulated in response to TLR3 and 9 stimulation (Figure 5 a, *p<*0.0001). VCAM-1 and ICAM-1 proteins were over-expressed in response to concomitant NP-CTL transfer and TLRs stimulation (Figure 5 b, e, *p* = 0,0013 and *p<*0.0001 respectively). The expression of Vap-1 remained unchanged at the transcription and protein levels for all conditions tested (Figure 5 g-i). Immunohistochemistry revealed the same expression pattern for VCAM-1, ICAM-1 and Vap-1 (Figure 5 c, f, i). ICAM-1 mRNA levels remained constant over the course of the experiment, while VCAM-1 levels increased steadily over time (*p*<0.0001 between 24 hrs and 8 days, Figure S1 and Figure 5 a, d). TLR3 and TLR9 stimulation and the presence of autoreactive T cells lead to an increased expression of adhesion molecules, thereby promoting inflammation of the liver.

### Apoptosis in the Liver

TUNEL-positive cells were found more frequently in mice that received TLR3 and 9 ligands alone or in association with NP-CTL transfer, confirming the bystander hepatitis caused by TLR stimulation (Figure 6 a and b, *p<*0.0001). The transfer of NP-CTL alone did not lead to a significant level of hepatocyte apoptosis. A significant increase of apoptosis was observed 72 hrs post-transfer (*p*<0.001, 24 hrs vs 72 hrs) which continued to increase up to 8 days after transfer (Figure 6 c, *p*<0.05, 72 hrs vs 8 days). Histological examination of liver sections revealed that when TLR3 and 9 ligands were administered, hepatocytes were TUNEL^+^ but liver-infiltrating cells were also dying (Figure 6 a).

### Fate of Adoptively Transferred Cells in the Liver

Serum ALT levels returned to normal 16 days after NP-CTL transfer and TLRs stimulation (Figure 7 a). While the proportion of Vβ12^+^ T cells (NP-CTLs bear Vβ12 chain) was not affected in the spleen at any time after transfer (data not shown), they were present in higher numbers in the liver 24 hrs and 72 hrs after transfer (Figure 7 b). Vβ12^+^ T cell numbers in the liver had declined by day 8 (Figure 7 b). CFSE-labeled NP-CTL cells were used to study the organ distribution and proliferation of adoptively transferred cells after TLR stimulation. IV-transferred NP-CTLs were preferentially recruited by the liver (24 hrs), proliferated until 72 hrs post-transfer but were no longer detectable by day 8 post-transfer, either due to CFSE dilution or cell deletion (Figure 7 c–d). However, the former is not likely, since the number of Vβ12^+^ T cells did not increase, but decreased to reach the baseline levels observed in the liver of untreated TTR-NP mice (Figure 7 b). CFSE^+^ cells were also found in the spleen 24 and 72 hours after transfer, but in lower numbers.

### Restoration of Immune Tolerance in the Liver

Flow cytometry analyses, using the viability marker 7-AAD, showed that there was a contraction of liver CD8^+^ T cells number (CD8^+^ 7-AAD^+^ cells) between day 3 and 8 (Figure 8 a, *p* = 0.0002, day 8 compared to control mice) and to a lesser extent in the spleen (Figure 8 a, *p* = 0.0007). Apoptosis of transferred cells (NP-CTLs) was detected 24 hrs after transfer and lasted at least until 72 hrs after transfer (Figure 8 b). Analyses revealed that apoptosis was induced by the intrinsic pathway (between 24 and 72 hours, Caspase 9^+^ cells, death by neglect) and by extrinsic pathways (72 hours post-transfer, Caspase 8 induction through death receptors) (Figure 8 c). Activation of Fas and FasL pathway can induce caspase 8 stimulation. In NP-CTL transferred mice stimulated with CpG and Poly(I:C), FasL was up-regulated from 72 hrs until day 8 in the liver (Figure 8 d, *p* = 0.0261). Fas expression in the liver was not significantly changed during that time period (Figure 8 e, *p* = 0.1539).

On day 8, mice injected with CpG and Poly(I:C) had higher frequencies of PD-1^+^ cells in the spleen and liver, which increased when autoreactive T cells were transferred (Figure 8 f, *p<*0.0001). PD-1 expression in lymphocytes occurred after NP-CTLs transfer, as virtually none of NP-CTLs were PD-1^+^ before transfer (1,1±1,3%, data not shown). The expression of PD-L1 was also up-regulated in the liver in response to the CpG and Poly(I:C) administration (Figure 8 g, *p<*0.0001), and was up-regulated from 72 hrs until day 8 post-transfer (Figure 8 h, *p* = 0.0093) suggesting that the PD-1/PD-L1 pathway could be involved in the restoration of immune tolerance [23].

## Discussion

Non-hepatotropic viral infections can lead to liver inflammation (ex: Epstein-Barr virus (EBV)), and have been frequently proposed as an etiological factor for autoimmune liver diseases (reviewed in [1]). A bystander hepatitis creating a fertile immunological environment for circulating autoreactive T cells could contribute to the initiation, and eventually the perpetuation of liver autoimmunity. The sole presence of autoreactive cytotoxic CD8^+^ T cells is not sufficient to break the liver immune tolerance and cause an autoimmune disease [3], [24], [25]. In fact, transfer of autoreactive T cells targeting a liver autoantigen results in anergy or antigen ignorance [24], [25]. Stimulation of TLR receptors can prevent this anergy and allow the development of an autoimmune response [24], [25]. Our work shows that beyond the prevention of anergy, stimulation of TLRs also results in the creation of a pro-inflammatory environment in the liver leading to the homing and recruitment of lymphocytes, and eventually to bystander hepatitis. However, while TLR 3 and 9 stimulation may be critical for promoting a liver autoimmune injury following transfer of autoreactive CTLs, it is not sufficient to induce a persistent autoimmune response. Indeed, lymphocytes, including autoreactive T cells, are recruited to the liver, proliferate and, when TLR stimulation stops, the liver rapidly restores tolerance by inducing their apoptosis. These results suggest that TLR3 stimulation may be more limited in its control of the immunoprivileged status of the liver than previously believed [3], and that the liver can restore immune homeostasis even after stimulation with strong pro-inflammatory signals and even in presence of autoreactive T cells.

TUNEL analyses performed in mice that received TLR3 and 9 ligands, with or without autoreactive T cells, revealed both apoptotic hepatocytes and lymphocytes. Apoptosis of hepatocytes could be the result of direct cytotoxicity from the autoreactive cells or other lymphocytes recruited to the liver, but also from direct toxicity of CpG and Poly(I:C), as suggested by the increase of TUNEL^+^ cells in liver of mice stimulated only with TLR3 and 9 ligands. In fact, direct stimulation of TLR3 on hepatocytes can induce apoptosis [26], and DNA released by dying hepatocytes is known to stimulate TLR9 on liver sinusoidal endothelial cells (LSEC) [27]. Thus, TLR3 and 9 stimulation can lead to bystander hepatitis. Bystander hepatitis is a phenomenon observed during systemic viral infections (reviewed in [28]). As we observed in our experimental model, this type of hepatitis is often self-limiting. Interestingly, we observed a similar self-limited hepatitis even in presence of autoreactive T cells. Liver-infiltrating lymphocytes were also TUNEL^+^, indicating that induction of cell apoptosis may be involved in the restoration of tolerance.

Transfer of autoreactive CD8^+^ T cells alone did not result in a significant liver injury, as suggested by the mildly elevated serum ALT levels and the low number of TUNEL^+^ hepatocytes. However, a low-grade liver inflammation was observed. The presence of autoreactive CTLs alone induced the over-expression of VCAM-1 and IP-10 that may have contributed to the recruitment of non-specific lymphocytes, explaining the inflammation observed on day 8. Also, the up-regulation of IP-10 suggests that INF-γ was over-expressed at some time earlier than day 8 post-transfer in response to autoreactive cells. Since lymphocytes in liver sections were not TUNEL^+^ and the expression of PD-L1 did not change following transfer, the restoration of the liver immune tolerance following autoreactive T cell transfer alone (in absence of strong innate immune stimulation) may not rely on apoptosis induction or PD-L1 expression.

The TLRs-induced pro-inflammatory environment was promoted by the over-expression of INF-γ and chemokines such as IP-10 (CXCL10), MIG (CXCL9) and CXCL16. The combination of INF-γ and CXCR3 ligands has proved to be essential for the recruitment of lymphocytes to a virus-infected liver, and binding of Fas promotes this recruitment [29]. Since FasL is up regulated in our model, cross-linking of Fas receptors on liver cells such as hepatocytes and LSEC maybe more frequent, promoting the secretion of IP-10, MIG and CXCL16.

TLR3 and 9 stimulation induced the over-expression of VCAM-1 and ICAM-1 in the liver. These molecules work together for the recruitment of lymphocytes in an antigen- dependant (ICAM-1) or -independent manner (VCAM-1), thus contributing to the bystander inflammation (VCAM-1) and autoreactive inflammation (ICAM-1) [30]. In contrast with previously published study on liver inflammation [31], levels of Vap-1 remained unchanged in our model. This difference could be explained by Vap-1 being predominantly associated with Th2-driven responses [32].

We propose that TLR9 stimulation, 24 hrs after T cell transfer, triggered a bystander hepatitis by first inducing INF-γ secretion in the liver. This up-regulation of INF-γ induced a strong up-regulation of CXCR3 ligands, IP-10 and MIG, two chemokines known to respond to γ-interferon signaling [33]. Recruitment of CXCR3^+^ cells by the liver (including transferred autoreactive T cells) took place before the recruitment of CXCR6^+^ cells. The bystander hepatitis induced by TLR3 and TLR9 stimulation produced a steady increase in the expression of VCAM-1. Increased expression of VCAM-1 was the result of TLR stimulation since mRNA levels were not influenced by the presence of autoreactive T cells. CXCL16 expression slowly increased after TLR3 and TLR9 stimulation. VCAM-1 and CXCL16 increased expression led to the recruitment of CXCR6^+^ cells by the liver, perpetuating the bystander hepatitis.

The observed bystander hepatitis and autoreactivity in this model are self-limiting suggesting that immune homeostasis is eventually restored in the liver. The combined induction of intrinsic (Bim) and extrinsic (Fas) pathway of apoptosis has been described as critical for immune homeostasis and CD8^+^ T cell contraction, but their combined action in the liver has been subject of discussions [34]. In our model, the liver immune tolerance was re-established by the elimination of CD8^+^ T cells through apoptosis induced via the intrinsic pathway (caspase 9 activation). This is consistent with previous reports, in which liver-infiltrating lymphocytes died by neglect, a process in which Bim plays a critical role as initiator of T-cell death [20], [35]. At 72 hrs post-transfer, lymphocytes also showed activated caspase 8, which may have been induced by death receptors activation such as Fas. In fact, FasL was up regulated in the liver, increasing apoptosis onset through death receptors ligation. Hepatocytes have the ability to use the Fas/FasL pathway to induce death in lymphocytes, and this ability is enhanced in presence of INF-γ[18]. The increase of both FasL and INF-γ in our model suggests that hepatocytes could take part in the elimination of transferred T cells. In addition, as a trigger of apoptosis, activation-induced cell death could be responsible for the deletion of the cells [36].

PD-1 and PD-L1 expression was induced in the liver 72 hrs post-transfer. While it cannot be excluded that their expression results from the activation of T cells [37], higher expression of PD-1/PD-L1 molecules are likely involved in the restoration of liver homeostasis. Several studies have shown that PD-1 and PD-L1 expression plays an important role in restoration of tolerance [23], [37], [38], [39]. PD-1/PD-L1 could contribute to the re-establishment of liver tolerance along with regulatory T cells (Tregs) and the induction of anergy or deletion of effectors T cells ([23] and reviewed in [37]). The latter may occur via LSEC, as the interaction of PD-1 on CD8^+^ T cells and PD-L1 on LSEC promotes tolerance in CD8^+^ T cells [39]. The involvement of PD-1 regulation in the control of CD8^+^ T cells function in the liver is consistent with previous reports using hepatitis-B virus transgenic mice [38]. In addition, PD-L1 has been described to be responsible for the accumulation and deletion of activated CD8+ T cells in the liver [23]. Other mechanisms could also be involved in the restoration of immune tolerance in the liver, including IL-10 [3], [40] and/or TGF-β secretion(s) [3], but further investigation is needed. Recently, another mechanism of immune tolerance in the liver, emperipolesis, was described [41]. During emperipolesis, autoreactive lymphocytes activated within the liver are degraded by hepatocytes [41]. However, in a liver pro-inflammatory environment, the role of emperipolesis in the elimination of lymphocytes activated in the periphery is still unknown.

In summary, in this model, transferred autoreactive T cells are preferentially recruited by the liver, in response to homing and recruitment signals up-regulated by TLR3 and 9 stimulation and the ensuing bystander hepatitis. Autoreactive cells proliferate until 72 hrs after transfer, at which point they begin to disappear. These observations are consistent with the notion that the liver eliminates antigen-triggered apoptotic cells *in vivo* [42], [43], specifically CD8^+^ T cells *via* ICAM-1 [42].

Requirements for a sustained break of liver immune tolerance in autoimmune liver diseases are still unknown. In the TTR-NP experimental model of AIH, liver injury is caused by autoreactive T cells primed in periphery of the liver by DNA vaccination with a plasmid coding for LCMV-NP, a neoantigen expressed by hepatocytes in TTR-NP mice [8]. This peripheral priming results in a proper activation [20] and proliferation of autoreactive CD8^+^ T cells and, as opposed to an adoptive transfer of autoreactive-CTLs, benefits from CD4^+^ T cells help [8], [10]. Presence of CD4^+^ T cell help could be an important element determining the final outcome of a liver autoimmune process. In the TTR-NP model of AIH, previous liver inflammation is not necessary, but the autoimmune liver disease is observed several months after DNA vaccination [8], [10]. This observation suggests that a constant and repeated assault of the liver by autoreactive CTLs may eventually overcome liver immune tolerance, especially in light of the fact that memory T cells are resistant to apoptosis in the liver [34]. An infiltration by autoreactive memory T cells as a way to break liver tolerance is consistent with the hypothesis that a long period of time could separate the initiating viral infection from the onset of autoimmune liver disease symptoms [1]. An important element of a chronic break of tolerance is the presence of genetic background permissive to the development of autoimmunity. In this study, the transgenic mice model used can develop a chronic autoimmune hepatitis [8]. Therefore, the transient nature of the hepatitis observed in this model is not due to the presence of limiting factors (central tolerance, etc.) but to the ability of the liver to restore immune homeostasis. Altogether, our findings show that a pro-inflammatory liver environment induced by TLRs stimulation results in bystander hepatitis, but it is not sufficient to maintain an autoreactive CTL response against the liver. The liver strong capacity to restore immune homeostasis is mediated in part through the apoptosis of infiltrating T cells and possibly through the promotion of the PD-1/PD-L1 pathway.

## Supporting Information

Figure S1 **mRNA Expression of Adhesion Molecules, Chemokines and Cytokine in the Liver at Different Time Post-transfer. A)** Adhesion molecules ICAM-1 and VCAM-1 mRNA expression. ICAM-1 expression stays stable over time while VCAM-1 expression increases (***p<0,0001 between 24 hrs and 8 days). **B)** Expressions of Interferon-γ (INF-g), IP-10 and MIG increase rapidly and peak on day 3 post-transfer. INF-γ, **p<0,001 24 hrs vs 72 hrs and *p<0,05 24 hrs vs 8 days; IP10, *** *p*<0,0001 24 hrs vs 72 hrs and 72 hrs vs 8 days; MIG, *** *p*<0,0001 for 24 hrs vs 72 hrs or 8 days and **p<0,001 for 72 hrs vs 8 days. CXCL16 expression slowly increases over time and is significantly higher on day 8 (***p<0,0001).
(PDF)

Table S1 **mRNA specific primers sequences used for RT-PCR.**
(PDF)
