# Supplementary figures and images for: Liver Restores Immune Homeostasis after Local Inflammation despite the Presence of Autoreactive T Cells
Source: PLoS One. 2012 Oct 24;7(10):e48192. doi: 10.1371/journal.pone.0048192 (PMC3480501; doi:10.1371/journal.pone.0048192)

Supplemental Figure S1.

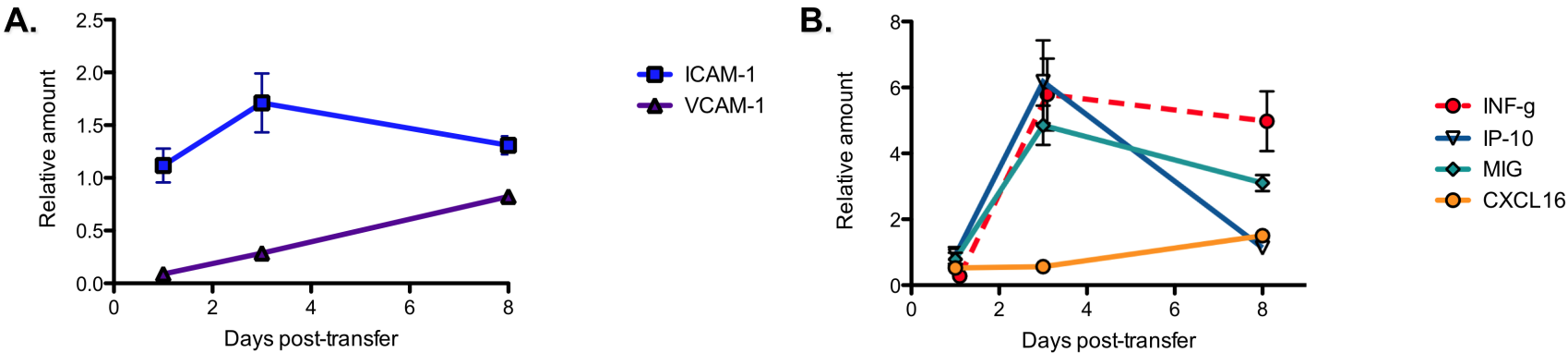

Supplement: Figure S1 — mRNA Expression of Adhesion Molecules, Chemokines and Cytokine in the Liver at Different Time Post-transfer. A) Adhesion molecules ICAM-1 and VCAM-1 mRNA expression. ICAM-1 expression stays stable over time while VCAM-1 expression increases (***p<0,0001 between 24 hrs and 8 days). B) Expressions of Interferon-γ (INF-g), IP-10 and MIG increase rapidly and peak on day 3 post-transfer. INF-γ, **p<0,001 24 hrs vs 72 hrs and *p<0,05 24 hrs vs 8 days; IP10, *** p<0,0001 24 hrs vs 72 hrs and 72 hrs vs 8 days; MIG, *** p<0,0001 for 24 hrs vs 72 hrs or 8 days and **p<0,001 for 72 hrs vs 8 days. CXCL16 expression slowly increases over time and is significantly higher on day 8 (***p<0,0001). (PDF) [file pone.0048192.s001.pdf]
