# Supplementary material for: Liver Restores Immune Homeostasis after Local Inflammation despite the Presence of Autoreactive T Cells
Source: PLoS One. 2012 Oct 24;7(10):e48192. doi: 10.1371/journal.pone.0048192 (PMC3480501; doi:10.1371/journal.pone.0048192)
Supplement: Table S1 — mRNA specific primers sequences used for RT-PCR. (PDF) [file pone.0048192.s002.pdf]

**TABLES**

**Supplemental Table S1. mRNA specific primers sequences used for RT-PCR**

| Gene | Primers sequence | mRNA product size |
|---|---|---|
| **TLR3** | Fwd: TTGTCTTCTGCACGAACCTG<br>Rev: CGCAACGCAAGGATTTTATT | mRNA=204pb |
| **TLR9** | Fwd: CCAGACGCTCTTCGAGAACC<br>Rev: GTTATAGAAGTGGCGGTTGT | mRNA= 318pb |
| **INF-α** | Fwd: ATGGCTAGGCTCTGTGCTTTCCT<br>Rev: AGGGCTCTCCAGATTTCTGCTCTG | mRNA=523pb |
| **INF-β** | Fwd: TCCTGCTGTGCTTCTCCACC<br>Rev: CCATCCAGGCGTAGCTGTTG | mRNA= 449pb |
| **INF-γ** | Fwd: TGGATATCTGGAGGAACTGGCA<br>Rev: TTTCGCCTTGCTGTTGCTGA | mRNA= 175pb |
| **ICAM-1** | Fwd: TCGGAGGATCACAAACGAAGC<br>Rev: AACATAAGAGGCTGCCATCACG | mRNA= 432pb |
| **VCAM-1** | Fwd: CCTCACTTGCAGCACTACGGGCT<br>Rev: TTTCCAATATCCTCAATGACGGG | mRNA= 441pb |
| **Vap-1** | Fwd : GGCGGCACCACTCAGATTTC<br>Rev : TGGGACTTTCCTGGGGCAAG | mRNA= 539pb |
| **IP-10** | Fwd: GTGCTGCCGTCATTTTCTGC<br>Rev: CCGGATTCAGACATCTCTGC | mRNA= 221pb |
| **MIG** | Fwd: GACATTCTCGGACTTCACTC<br>Rev: GATTCAGGGTGCTTGTTGGT | mRNA= 467pb |
| **CXCL16** | Fwd: GCTTTGGACCCTTGTCTCTTGC<br>Rev: GTGCTGAGTGCTCTGACTATGTGC | mRNA= 409pb |
| **FasL** | Fwd: CAGCTCTTC CAG CTG CAG AAG G<br>Rev: AGATTCCTCAAAATTGATCAGAGA GAG | mRNA=509pb |
| **Fas** | Fwd: AGCCGTGCACAGCAACAACCAGCA<br>Rev:AGCAGCTGGACTTTCTGCTCAGC | mRNA=358pb |
| **PDL1** | Fwd: AAGCCTCAGCACAGCAACTT<br>Rev: ACATCATTCGCTGTGGCGTT | mRNA= 392pb |
